# The role of spatial texture in visual control of bumblebee learning flights

**DOI:** 10.1007/s00359-018-1274-0

**Published:** 2018-07-06

**Authors:** Nellie Linander, Marie Dacke, Emily Baird, Natalie Hempel de Ibarra

**Affiliations:** 1 Lund Vision Group, Department of Biology, Lund University, Lund, Sweden; 2 Centre for Research in Animal Behaviour, Psychology, University of Exeter, Exeter, EX4 4QG UK

**Keywords:** Flight control, Flight height, Ventral optic flow, Bees, Insects

## Abstract

When leaving the nest for the first time, bees and wasps perform elaborate learning flights, during which the location of the nest is memorised. These flights are characterised by a succession of arcs or loops of increasing radius centred around the nest, with an incremental increase in ground speed, which requires precise control of the flight manoeuvres by the insect. Here, we investigated the role of optic flow cues in the control of learning flights by manipulating spatial texture in the ventral and panoramic visual field. We measured height, lateral displacement relative to the nest and ground speed during learning flights in bumblebees when ventral and panoramic optic flow cues were present or minimised, or features of the ground texture varied in size. Our observations show that ventral optic flow cues were required for the smooth execution of learning flights. We also found that bumblebees adjusted their flight height in response to variations of the visual texture on the ground. However, the presence or absence of panoramic optic flow did not have a substantial effect on flight performance. Our findings suggest that bumblebees mainly rely on optic flow information from the ventral visual field to control their learning flights.

**Electronic supplementary material:**

The online version of this article (10.1007/s00359-018-1274-0) contains supplementary material, which is available to authorized users.

## Introduction

Like most hymenopteran insects, bees search for food far away from their nest. From here, they then need to find their way back to their hive. Some insects meet the challenge of homing by leaving pheromone trails (for example, several ant species, for a review see Jackson and Ratnieks 2006), while other walking and flying insects such as desert ants, wood ants, wasps and bees primarily rely on visual cues for navigation (for reviews see Collett et al. 2006; Warrant and Dacke 2011; Zeil 2012; Webb and Wystrach 2016). When leaving the nest for the first time, bees and wasps perform learning flights to memorise the location of the nest (e.g. Tinbergen 1932; Zeil 1993a, b; Hempel de Ibarra et al. 2009; for a review see Zeil et al. 1996). By positioning themselves at different angles and distances from the nest, these insects acquire views of the nest and its surroundings, which are subsequently used for locating the nest entrance during return flights (Philippides et al. 2013; Robert et al. 2018; Stürzl et al. 2016; for reviews see; Zeil et al. 1996; Collett et al. 2016). The complex flight patterns seen during these elaborate flights must be controlled in a very precise manner, but not much is known about what visual information they use to do this. Here, we focus on the importance of cues extracted from optic flow fields, which are known to mediate visually guided flight control in insects for various types of flight.

To control cruising flight, insects extract information from the apparent translational image motion that is generated on the retina as they move through the world, known as optic flow (Gibson 1950, 1979). During forward motion, the magnitude of translational optic flow varies with the distance to nearby surfaces so that closer objects appear to move faster than those that are further away, creating a vector field all around the insect. Thus, the pattern of optic flow can provide important information about an animal’s self-motion and the spatial layout of the environment (Koenderink 1986; Lappe 2000; Collett 2002). Animals are highly sensitive to optic flow cues but using them depends upon the visual system’s capacity to resolve contrast differences in the visual scene. Translational front-to-back optic flow cues are used by honeybees, bumblebees and fruit flies to control their ground speed (David 1982; Srinivasan et al. 1996; Baird et al. 2005, 2010; Barron and Srinivasan 2006; Fry et al. 2009; Portelli et al. 2011; Linander et al. 2015, 2016). Ventral front-to-back optic flow cues—which depend upon the ratio of ground speed over height above the ground—are also used by honeybees, bumblebees and flies to control their height above the ground (Baird et al. 2006; Portelli et al. 2010, 2017; Straw et al. 2010; Linander et al. 2016).

The requirements for controlling a learning flight, however, differ from cruising flight in that the insect actively varies the components of its flight trajectory to facilitate the extraction of visual information to learn the location of the nest.

During learning flights, wasps fly in a very characteristic pattern of continuously expanding arcs where height, lateral displacement and ground speed continuously increase (for a review see Zeil et al. 1996). This keeps the insect flying within a cone of space that extends away and upwards from the nest entrance to an approximate height of 20 cm above the ground (Stürzl et al. 2016). By pivoting around the goal in this quite stereotyped manner, the insect keeps the nest within defined regions of its visual field throughout the flight, which simplifies the learning process. The arc-shaped flight pattern also creates a motion parallax centred around the nest that can be used to estimate the distance to various landmarks in relation to the nest (Zeil 1993a, 1997; Riabinina et al. 2014).

In comparison with wasps, the learning flights of bumblebees are much more variable. The most repeatable sections of their learning flights are loops interspersed with segments of straight flight (Collett et al. 2013; Philippides et al. 2013). Whilst variable in size and shape, loops increase in diameter over the duration of the flight and they usually end at, or close to, the nest (Philippides et al. 2013). During their learning flights, bumblebees face the nest many times, memorising views that guide them back to the location of the nest (Hempel de Ibarra et al. 2009; Collett et al. 2013; Philippides et al. 2013; Robert et al. 2017, 2018). Recordings of learning flights under natural conditions show that they achieve this by actively adjusting their body and head orientations (which are closely associated), with the result that these often diverge from the flight direction (Philippides et al. 2013; Riabinina et al. 2014). Thus, during a learning flight, bumblebees not only fly forwards but they also pivot and turn, move sideways and display very brief instances of hovering or even backwards flight. The question arises what information the bees use to control these elaborate flight patterns. Although bees and wasps can use translational optic flow to extract information about spatial layout of the nest surroundings (Zeil 1997; Dittmar et al. 2010; Mertes et al. 2014; Riabinina et al. 2014), its role in controlling the complex manoeuvres performed during learning flights remains to be studied.

The aim of the present study was to investigate whether bumblebees use translational optic flow to control the fine movements of the learning flight. We recorded learning flights of bumblebees (*Bombus terrestris*) in an artificial environment where we manipulated the spatial texture, and thus the availability of optic flow cues, in the bee’s ventral and panoramic field of view. We measured how these manipulations affected features of the learning flight such as height, ground speed and lateral distance from the nest. Overall, we find that, as in cruising flight, ventral optic flow cues play an important role in the control of learning flights.

## Materials and methods

### Experimental set-up

**Fig. 1:**
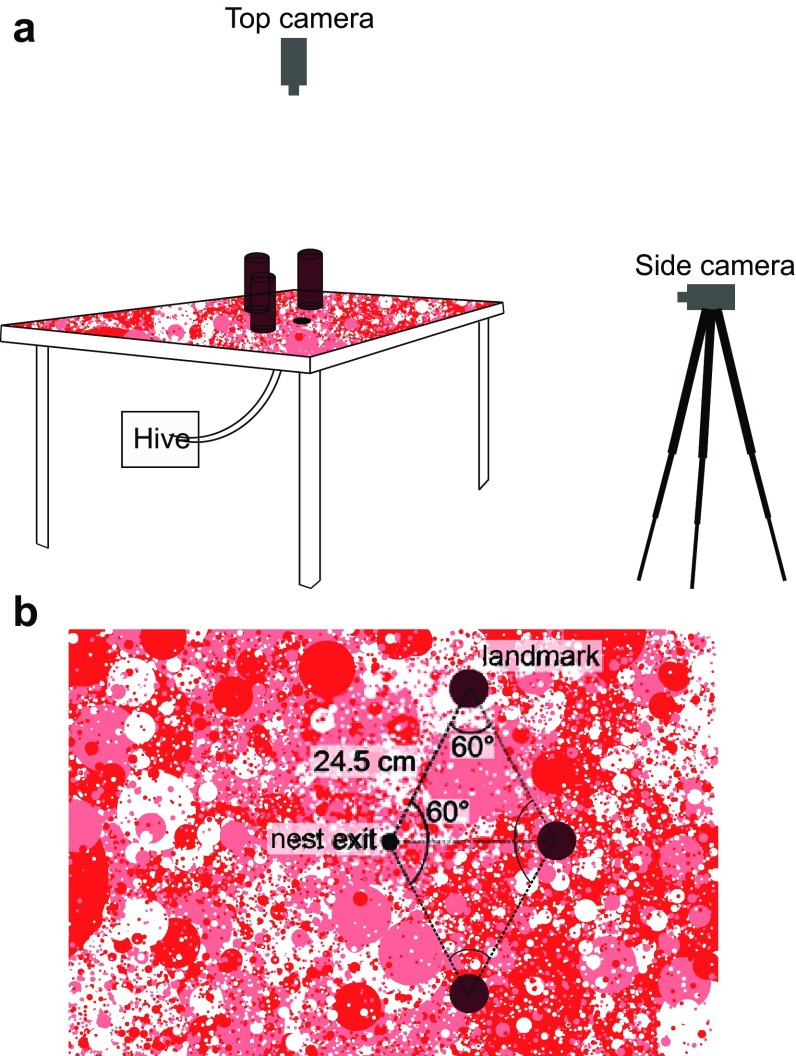
Experimental set-up. **a** Sketch of the experimental set-up. Bees emerged from a nest exit on the top of a platform. Flights were recorded continuously at 50 fps with two cameras, one with a top view of the platform (centred on the nest exit) and the other with a side view of the platform. **b** Top view of the experimental platform (140 × 150 cm) showing the arrangements of the landmarks (5.5 cm diameter, 20 cm high) around the nest exit (2.5 cm diameter). The landmarks were placed at equal angles (60°) and distances (24.5 cm) from the nest exit and from each other

Experiments were conducted in a temperature-controlled room (3.5 × 5 m, 3.7 m height; 21 °C) at the University of Exeter, UK. Bumblebees (*B. terrestris audax*, Koppert UK) emerged from a nest exit (2.5 cm diameter) on the top of a table (140 × 150 cm, 92 cm height) placed in the centre of the experimental room. Three dark red vertical cylinders (5.5 cm diameter, 20 cm high) were placed at equal angles (60°) and distances (24.5 cm) from the nest exit and from each other (Fig. 1). To vary the optic flow available in the ventral field of view, the experimental platform was covered with four different patterns; 1 × 1 mm, 5 × 5 mm and 10 × 10 mm dark red and white checkerboard pattern and a red ‘dead leaf pattern’ (DL) (see also Table 1). While the checkerboard patterns would limit the height at which bumblebees could resolve the texture and, therefore, experience optic flow cues in the ventral visual field, the dead leaf pattern was designed to make optic flow estimations distance independent (for technical specifications see Lee et al. 2001). The patterns presented strong contrasts (Michelson contrast: checkerboard patterns 0.6, DL pattern 0.41–0.62).

**Table 1:** Experimental conditions

Condition	Experimental platform	Walls
1 mm-P	1-mm check pattern	Dead leaf pattern
5 mm-P	5-mm check pattern	Dead leaf pattern
10 mm-P	10-mm check pattern	Dead leaf pattern
DL-P	Dead leaf pattern	Dead leaf pattern
1 mm-W	1-mm check pattern	White
5 mm-W	5-mm check pattern	White
10 mm-W	10-mm check pattern	White
DL-W	Dead leaf pattern	White

Each bee was tested only once on her first departure from the nest

The spatial resolution in *B. terrestris* is 0.21 cycles deg^−1^ when tested in a Y-maze experiment (Chakravarthi et al. 2016) and between 0.19 and 0.23 cycles deg^−1^ (Chakravarthi et al. 2017) when tested during cruising flight. From this data, it follows that the bumblebees can resolve two points with an angular separation of approximately 5.3° (1/0.19), and thus we estimated that the 1-mm-large checkerboard pattern would be resolved by the bees up to 2 cm above the surface of the platform (*x* = tan (5.3) × 20 mm; where *x* is the size of one black and one white square). The corresponding values for the 5- and the 10-mm-large checkerboard patterns are 10 cm (mid-height of the cylinders) and 20 cm (top-height of the cylinders), respectively. To vary the optic flow available in the panoramic field of view, the walls of the experimental room were either completely white (W) or lined with a dead leaf pattern (P). Flights were recorded for eight experimental conditions; four with varying patterns on the surface of the platform and the dead leaf pattern on the walls (1 mm-P, 5 mm-P, 10 mm-P and DL-P) and four where the walls were white (1 mm-W, 5 mm-W, 10 mm-W and DL-W). Five hives were used throughout the experiments, and for as long as the hives contained active foragers they were used for all conditions, thus preventing any effects of colony on the results. For each condition, a sample size of approximately 30 bees (between 27 and 32) was used. Each bee was tested only once, and was, therefore, naive to the visual surroundings of the nest-exit. After the completion of the learning flight, each individual was captured and removed from the experiment. Therefore, it was not necessary to mark them individually.

Flights were recorded continuously at 50 fps with two cameras (Sony HDR CX410, Tokyo, Japan), one with a top view of the platform (centred on the nest exit, 1.6 m above the platform) and the other with a side view of the platform (2.75 m from the nest exit, at a height of 100 cm). The zoom settings and position of both cameras were fixed. Using calibration patterns, we checked for optical distortions that would require corrections of the trajectory coordinates, but did not find any within the recording area and up to a height of 20 cm. The flight of each bee was recorded until it left the field of view of the top camera, which covered an area of 133 cm × 75 cm. If the bee left the recording area for more than 1 s, its learning flight was considered terminated. Thus, the duration of a learning flight was determined by the time each bee spent in the recording area. It should be noted that the bees mainly performed their learning flights within the landmark zone (see Fig. 1b), and they rarely came back after leaving the recording area the first time.

### Data analysis

The centre of mass of the bumblebee was determined (in *x*- and *y*-pixel coordinates) in each video frame using an automated tracking programme (Lindemann 2005). Positions were checked by eye and corrected if necessary. Data were converted from pixels to cm using a reference checkerboard pattern (1 × 1 cm check size), placed at three different locations. The height and lateral position of the bee was calculated with respect to the nest (0, 0 coordinate). Given that the camera positions and zoom settings remained unchanged throughout the experiment, it was not necessary to correct the trajectories for depth distortion. The height distribution for each flight was computed from the recordings of the synchronised side camera. Since the learning flights were very variable and flight occurrences above the landmarks turned out to be rare, analyses only included data below the height of the landmarks (< 20 cm). Lateral distance from the nest was computed from the recordings of the top camera. Ground speed, also analysed from the top camera, was determined by calculating the two-dimensional distance travelled between each frame and dividing this by the time step between the frames. The length of a flight trajectory, referred to as path length, was calculated by adding the total distance in *x*- and *y*-coordinates that a bee has flown. The data were analysed in 2D, i.e. separately for the side camera and for the top camera. Corresponding flights from the two cameras were synchronised with a lab clock (tenth of a second precision) made visible in both cameras by projecting a mirror image of the clock into the field of view of the second camera between each flight. This procedure enabled us to perform correlation analysis between different flight control parameters, such as, for example, height and ground speed.

Since the data were not normally distributed, a non-parametric Wilcoxon rank-sum statistical test at the 5% significance level was used to analyse the data, and a Bonferroni correction was applied when multiple comparisons were made on the same data set. Pearson’s linear correlation coefficient (*r*) was calculated to test for a correlation between different variables within a flight.

### Evaluation of flight performance

It could be expected that removal or manipulation of spatial texture would disrupt the bumblebee’s ability to execute well-controlled learning flights. It is indeed easy to observe when a bee has significant difficulties in producing the elaborate flight manoeuvres that are characteristic of a learning flight. Even though the learning flights of bumblebees are variable, both within and between individuals, they are easily recognised by their steady loop-shaped trajectories. Initially, the flight is slow, and the bee hovers or flies very close to the nest; later in their flight it steadily increases flight speed and its distance from the nest (Hempel de Ibarra et al. 2009; Collett et al. 2013; Philippides et al. 2013; Robert et al. 2017, 2018). Bumblebee learning flights are particularly elaborate and long when the bee leaves the nest for the first time and, in our experiment, only the first learning flight of each bee was analysed. A learning flight that is poorly executed will manifest itself, for example, when the bee departs very quickly, without the typical initial slow-flight phase centred around the nest (Robert et al. 2018). Additionally, such flights often take the bee away from the nest for most of the flight and lack the characteristic steady loops that increase as the flight progresses. Consequently, they are easy to distinguish in real time. It should be also noted that poorly executed flights still differ from the very straight departure flights of experienced workers or when male bumblebees leave the natal nest (Robert et al. 2017). In addition to qualitative observations, we expected to measure large standard deviations around the mean ordinate for parameters such as height and lateral distance from the nest if bees had difficulties in controlling their learning flights in one of the test conditions.

## Results

### Learning flight performance with panoramic and ventral optic flow cues present

**Fig. 2:**
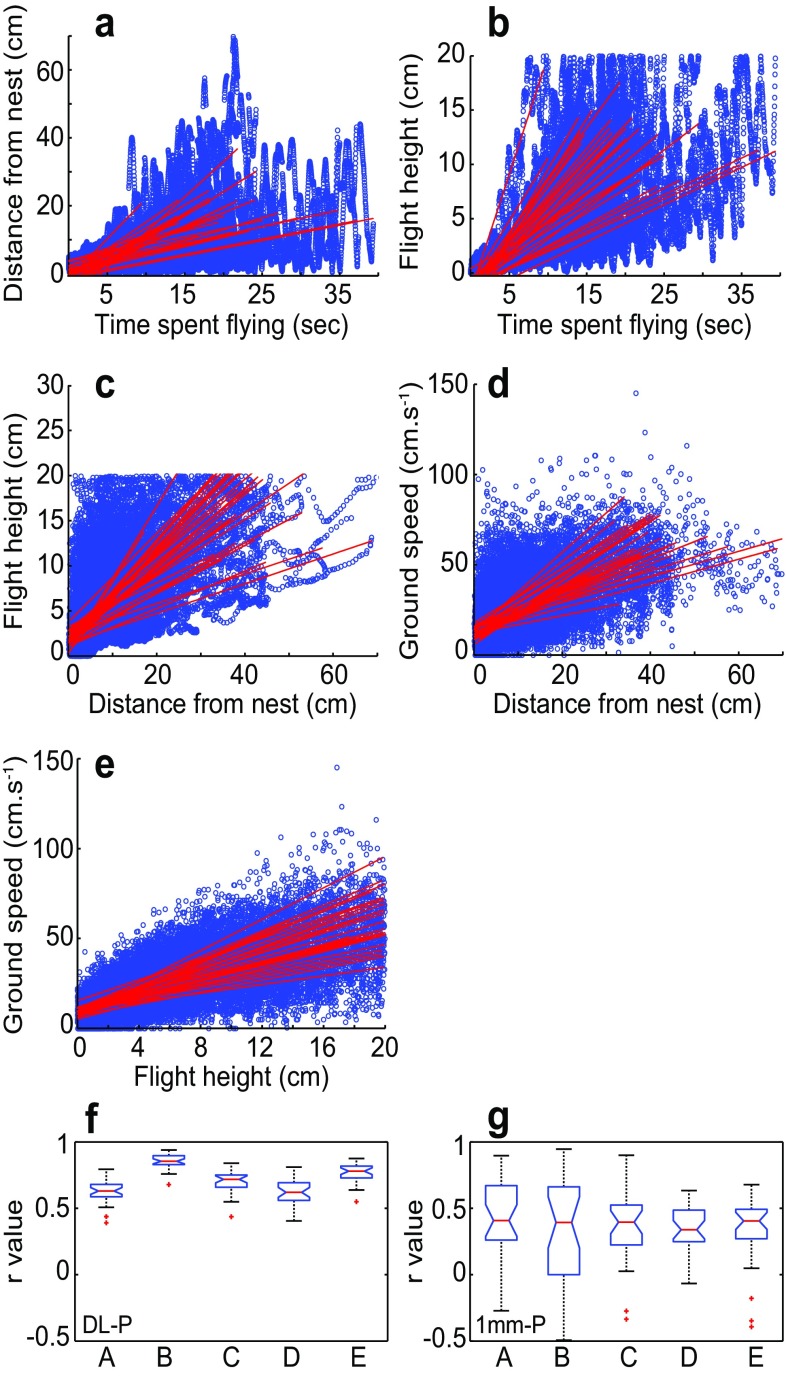
Flight control in the presence of strong ventral and panoramic optic flow cues. Subplots *a*–*f* represent condition DL-P (*n* = 31) (dead leaf pattern on the experimental platform, and dead leaf pattern on the walls), and subplot *g* represents condition 1 mm-P (*n* = 29) (1-mm check pattern on the experimental platform, and dead leaf pattern on the walls). **a** Lateral distance from the nest as a function of time spent flying. **b** Flight height as a function of time spent flying. **c** Flight height as a function of lateral distance from the nest. **d** Ground speed as a function of lateral distance from the nest. **e** Ground speed as a function of height flown above the surface of the platform. Red lines indicate a linear regression fit to the data for each flight. **f, g** The associated mean of the Pearson’s linear correlation coefficients (*r*) for condition DL-P (**f)** and 1 mm-P (**g**). Note that A–E on the *x*-axis in subplots *f* and *g* correspond to subplots *a*–*e*, respectively. Blue boxes indicate the extent of the 25–75% interquartile range, the red horizontal line in the box indicates the median, whiskers indicate the full extent of the data and red crosses represent outliers

In the presence of rich optic flow information in the ventral and panoramic visual fields (dead leaf pattern, condition DL-P), bumblebees conducted typical learning flights, as expected. Initially, the bees flew in small loops that were concentrated in a small area around the nest exit. Later on in flight, larger loops followed as the bees increased their distance moving away from the nest. Lateral distance from the nest increased as the flight progressed (*r* = 0.63 ± 0.1, Fig. 2a, f), which indicates that the loops of the learning flight became larger and stretched further away from the nest the longer the bees had been flying. This is consistent with earlier reports in bumblebees (Collett et al. 2013; Philippides et al. 2013). Flight height also increased over time (*r* = 0.85 ± 0.05, Fig. 2b, f), although the relationship for each individual was not linear as the bees often varied their flight height during the learning flight. As the bees flew further away from the nest, they also increased their flight height (*r* = 0.70 ± 0.09, Fig. 2c, f) and ground speed (*r* = 0.63 ± 0.09, Fig. 2d, f). The latter is in agreement with earlier reports in wasps and bumblebees (e.g. Zeil 1993a; Philippides et al. 2013). Similar to a study by Linander et al. (2016) investigating cruising flight in bumblebees, we found a correlation between height and ground speed (*r* = 0.77 ± 0.07, Fig. 2e, f), which indicates that optic flow cues in the ventral field of view are also important for flight control in learning flights.

### Learning flight performance with optic flow cues present or absent

**Fig. 3:**
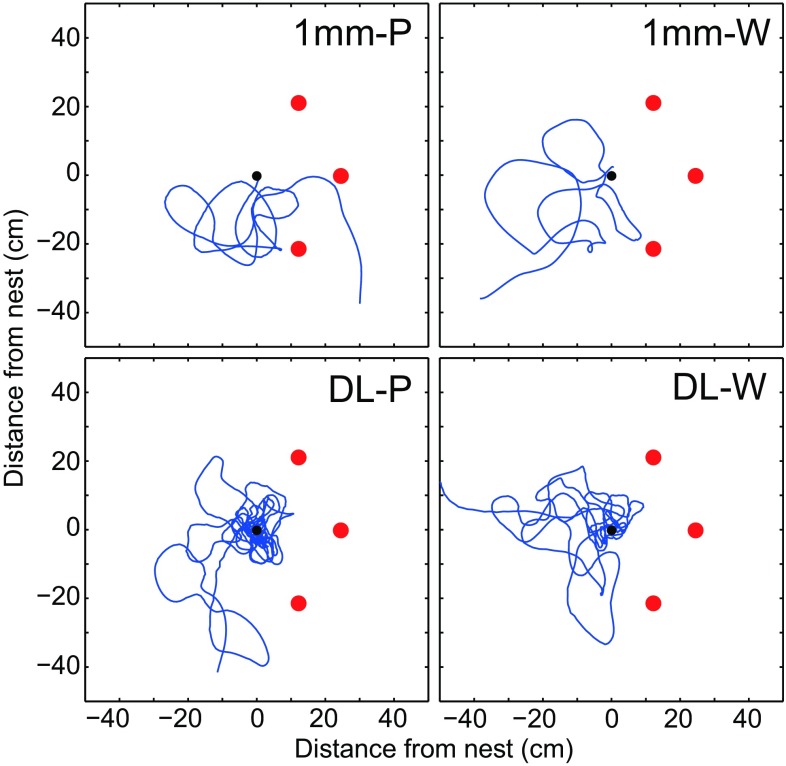
Examples of flight trajectories as viewed from above. The walls were either lined with dead leaf pattern (left column:1 mm-P, DL-P), or completely white (right column; 1 mm-W, DL-W). The upper row shows trajectories for a bee flying over a pattern (1-mm checks) only generating ventral optic flow at the surface of the platform, but no ventral optic flow after take-off. The lower row shows trajectories for a bee flying over a dead leaf pattern (DL) which generates rich ventral optic flow cues during the whole flight. The condition is specified in the top right corner of each plot. The red dots mark the position of the dark-red cylinders that surrounded the nest exit (black dot)

**Fig. 4:**
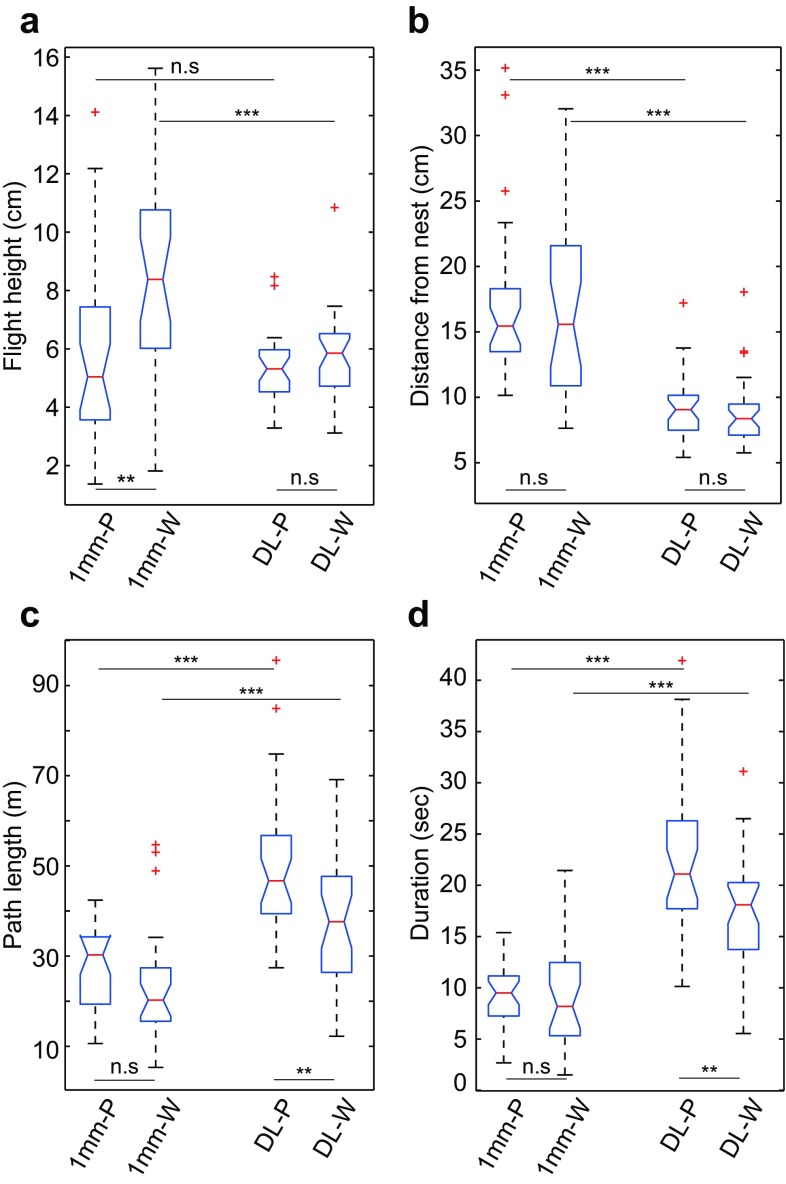
The effect of optic flow on the control of learning flights. **a** Average flight height above the surface of the platform. **b** Average lateral distance from the nest. **c** Average path length. **d** Average flight duration. Blue boxes indicate the extent of the 25–75% interquartile range, the red horizontal line in the box indicates the median, whiskers indicate the full extent of the data and red crosses represent outliers. Black stars indicate the level of significance (Wilcoxon rank-sum): **p < 0.01, ***p < 0.001. *n.s*. not significant (*p* > 0.025). The condition is specified on the *x*-axis: 1 mm-P = 1 mm check pattern on the experimental platform, and dead leaf pattern on the walls (*n* = 29). 1 mm-W = 1 mm check pattern on the experimental platform, and white walls (*n* = 27). DL-P = dead leaf pattern on the experimental platform, and dead leaf pattern on the walls (*n* = 31). DL-W = dead leaf pattern on the experimental platform, and white walls (*n* = 32)

Next, we investigated the bees’ ability to control their learning flight when ventral optic flow cues were minimised. Flights over the dead leaf pattern (DL) were compared with flights over a pattern with a texture that is too fine for the bees to resolve after take-off (1-mm-large checks). Over the dead leaf pattern, both in the presence (DL-P) and in the absence (DL-W) of panoramic optic flow cues, the bumblebees conducted typical learning flights consisting of well-concentrated loops around the nest (Fig. 3, lower row). Over the 1-mm checks, the bees were moving more erratically, sliding from side to side, which clearly showed severe disruption of their ability to control the learning flight (Fig. 3, upper row). This conclusion is supported by the large variation in flight height (Fig. 4a) and in the distribution of lateral positions around the nest (Fig. 4b), in the learning flights over the 1-mm checks (see also Fig. S2). Moreover, bumblebees flew further away from the nest in the 1-mm check condition (1 mm-P vs. DL-P: *Z* = − 6.29, *p* < 0.001; 1 mm-W vs. DL-W: *Z* = − 4.37, *p* < 0.001, Fig. 4b), their flight trajectories were shorter (1 mm-P vs. DL-P: *Z* = −5.47, *p* < 0.001; 1 mm-W vs. DL-W: *Z* = − 3.80, *p* < 0.001, Fig. 4c) and they flew over the platform for a shorter period of time (1 mm-P vs. DL-P: *Z* = − 6.29, *p* < 0.001; 1 mm-W vs. DL-W: *Z* = − 4.37, *p* < 0.001, Fig. 4d). Similar behaviours can also be observed when departing bumblebees fly over completely white surfaces (unpublished observations by Hempel de Ibarra N, Philippides A and Collett TS). Altogether, these results suggest that ventral optic flow does play a role in the control of learning flights.

This, however, raises the question of the importance of spatial texture in the panoramic visual field for the control of learning flights. In the next set of experiments, the learning flights of a new group of bees were recorded when the optic flow cues from the panoramic field of view were minimised (white walls, condition DL-W) while strong optic flow cues were present in the ventral field of view (dead leaf pattern, DL-P). In the absence of panoramic optic flow cues, the bees controlled their height and lateral distance from the nest in the same way as when these cues were present (Fig. 4a, b) although both the path length (*Z* = 2.87, *p* < 0.01) and duration (*Z* = 2.87, *p* < 0.01) of the DL-W flights were significantly shorter (Fig. 4c, d). Hence, the loss of panoramic optic flow seems to affect the complexity (path length) and thereby also the duration of the learning flight.

### Varying the availability of ventral optic flow

**Fig. 5:**
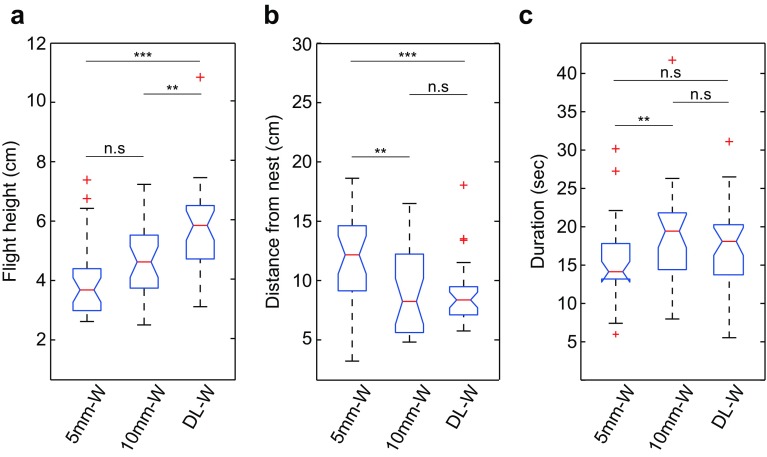
Impact of loss of ventral optic flow at different flight height. The subplots show the bumblebee response when ventral optic flow is only available up until a certain height (limited by the ability of the bees to resolve the pattern on the experimental platform). The walls were white (W). The condition is specified on the *x*-axis: 5 mm-W = 5 mm check pattern on the experimental platform, ventral optic flow is not resolvable above 10 cm (*n* = 29). 10 mm-W = 10 mm check pattern on the experimental platform, ventral optic flow is not resolvable above 20 cm (*n* = 29). DL-W = dead leaf pattern on the experimental platform, ventral optic flow is resolvable throughout all flight heights (*n* = 32). **a** Average height flown above the surface of the platform. **b** Average lateral distance from the nest. **c** Average flight duration. Boxes indicate the extent of the 25–75% interquartile range, the horizontal line in the box indicates the median, whiskers indicate the full extent of the data and red crosses represent outliers. Black stars indicate the level of significance (Wilcoxon rank-sum): **p < 0.01, ***p < 0.001. *n.s*. not significant (*p* > 0.025)

Since ventral optic flow cues appear to play an important role in the control of bumblebee learning flights, we further investigated how the bees respond when ventral optic flow was resolvable only up to a certain height. We covered the experimental platform with either 5- or 10-mm check patterns, so that the bumblebees could perceive it up to a height of 10 and 20 cm, respectively. The walls were kept white to minimise the effect of panoramic optic flow cues. Overall, we found that mean flight height decreased as the size of the checks in the ventral visual field decreased (Fig. 5a). Bumblebees flew lower over both the 5 mm (*Z* = − 4.37, *p* < 0.001) and the 10-mm check patterns (*Z* = − 3.17, *p* < 0.01) compared to when the dead leaf pattern was in the ventral field of view (DL-W). They also tended to fly lower over the 5-mm checks than over the 10-mm checks, although this was not statistically significant after correcting for multiple comparisons (*Z* = − 2.12, *p* = 0.034). Nevertheless, in all three conditions the learning flights were concentrated at heights below 10 cm, suggesting that bumblebees preferentially conduct their learning flights close to the ground.

When analysing the horizontal distance from the nest, the results showed that the learning flights were concentrated closer to the nest over the dead leaf pattern than when they flew over the 5-mm-large checks (*Z* = 3.50, *p* < 0.001, Fig. 5b). This suggests that bees tended to drift further from the nest when they reached heights where they could no longer resolve the pattern in the ventral field of view. Accordingly, the flights were also more concentrated around the nest over the 10-mm checks compared to the 5-mm checks (*Z* = 2.92, *p* < 0.01, Fig. 5b).

When comparing the total duration of the learning flights (as determined by the time each bee spent in the recording area) under the different conditions of ventral optic flow, flight duration did not vary systematically, suggesting that the bees could probably conduct sufficiently long learning flights under all conditions (Fig. 5c).

To summarise, bumblebees appeared to adjust their flight height to maintain sufficient ventral optic flow input. This indicates that bumblebees do use ventral optic flow cues to control their learning flights. Similar results are obtained when panoramic optic flow cues are present (see Supplementary Fig. S1 in Online Resource 1) and we found no evidence for interactions between ventral and panoramic optic flow that would influence flight control (see Supplementary Table S1 in Online Resource 1).

## Discussion

When presented with rich optic flow cues in both the ventral and the panoramic field of view (condition DL-P), bumblebees conducted well-controlled learning flights that were focussed on the nest exit (Figs. 3, 4b). Whilst initially staying close to the nest exit, the later parts of the flight took the bumblebees further away (Fig. 2a, b), where they also tended to fly faster and higher (Fig. 2d, e). Thus, the bees appeared to hold the magnitude of ventral optic flow around a desired set point by increasing ground speed as they gained altitude. By maintaining a constant rate of translational optic flow, ground speed and altitude will be automatically adjust to fit the spatial layout of the environment (Srinivasan et al. 1996; Baird et al. 2005, 2006, 2010; Franceschini et al. 2007). When the ventral texture was not resolvable after take-off (1 mm-P and 1 mm-W), flight height and lateral position around the nest exit was more variable (Fig. 4a, b), the duration and path length of the flights were much shorter (Fig. 4c, d) and there was no correlation between flight height and ground speed (Fig. 2g). Altogether, these results suggest that ventral optic flow is important for bees to conduct well-controlled flights, both when cruising towards a food source (Baird et al. 2006; Portelli et al. 2010; Linander et al. 2016, 2017) as well as while performing learning flights.

The absence of panoramic optic flow did not have a substantial effect on the bumblebees’ ability to conduct their learning flights or to control flight height, ground speed and lateral position around the nest (Fig. 4a, b). The shorter duration and path length of the flights (Fig. 4c, d) hint at the possibility that the presence of panoramic optic flow might enable the bees to modulate the flight trajectories further. It might be important for the fine-scaled control of the different manoeuvres during learning flights but consequently also for learning, potentially enhancing the bee’s capacity to actively acquire views around the nest. The analysis of trajectories undertaken in the present study reveals that bumblebees increase their flight height with distance from the nest (Fig. 2c), suggesting that they might fly in loops that take them not only further away from the nest in the horizontal plane but also in the vertical plane. During the learning flight, the bees constantly change their flight height and their distance from the nest (i.e. flying up and down, back and forth from the nest) (Fig. 2a, b). These recurrent changes in height and distance from the nest might be important features of the learning flight, aiding the bees to view the nest from different distances and angles. However, to fully understand the functional consequences of the complexity of bumblebee learning flights, we need to gain a deeper understanding of their 3D structure under natural conditions. Further studies analysing bumblebee learning and return flights in 3D could reveal interesting functional adaptations that facilitates the active acquisition of visual information.

When the availability of ventral optic flow cues was severely limited by the fineness of the texture on the ground, the bumblebees appeared to adjust the overall altitude of the learning flight to a level that would enable them to resolve the ventral optic flow cues (Fig. 5a). This is similar to observations made for the control of cruising flight in honeybees (e.g. Portelli et al. 2010), and suggests that the main input of optic flow required for the control of learning flights is obtained in the ventral field of view. Although we did not investigate the functional consequences of these adjustments (for example, whether they affected the learning of the nest position), it seems that bees are able to adapt their learning flights to maximise the detectability of ventral optic flow cues throughout the flight. This demonstrates once more the flexibility that bees exhibit during these flights. Furthermore, we found no differences in the duration of the learning flights over the different ground textures (Fig. 5c), which indicate that the bees completed their learning flights despite these variations in ventral optic flow. Our findings are relevant for understanding how bumblebees learn the unpredictable locations of their nest in different habitats (e.g. Fussel and Corbet 1992).

In summary, our results suggest that bumblebees use cues derived from ventral and panoramic image motion to accurately control learning flights. More specifically, we show that the presence of ventral optic flow cues is important, and that bumblebees adjust their flight manoeuvres to maintain continuous ventral optic flow input. In the absence of ventral optic flow cues, the flights become more variable in terms of flight height and lateral distance from the nest, and the typical looping pattern disappears. Whether panoramic optic flow cues are present or not does not strongly affect the overall structure of the learning flight, but these cues might still be involved in fine-scale flight control. Finally, we found that, when the availability of ventral optic flow was limited to certain heights, bumblebees showed flexibility in their behaviour by adjusting their flight height. This suggests that bees are able to cope with a range of ground textures when conducting learning flights in different visual environments.

## Electronic supplementary material

Below is the link to the electronic supplementary material.

Supplementary material 1 (PDF 4593 KB)
