# Supplementary material for: The role of spatial texture in visual control of bumblebee learning flights
Source: J Comp Physiol A Neuroethol Sens Neural Behav Physiol. 2018 Jul 6;204(8):737–45. doi: 10.1007/s00359-018-1274-0 (PMC6096632; doi:10.1007/s00359-018-1274-0)
Supplement: Supplementary file 1 — Supplementary material 1 (PDF 4593 KB) [file 359_2018_1274_MOESM1_ESM.pdf]

Supplementary material

The role of spatial texture in visual control of bumblebee learning flights

Journal of Comparative Physiology A

Linander N*, Dacke M, Baird E, Hempel de Ibarra N*

*Corresponding authors: nellie.linander@biol.lu.se, n.hempel@exeter.ac.uk
Lund Vision Group, Department of Biology, Lund University, Sweden

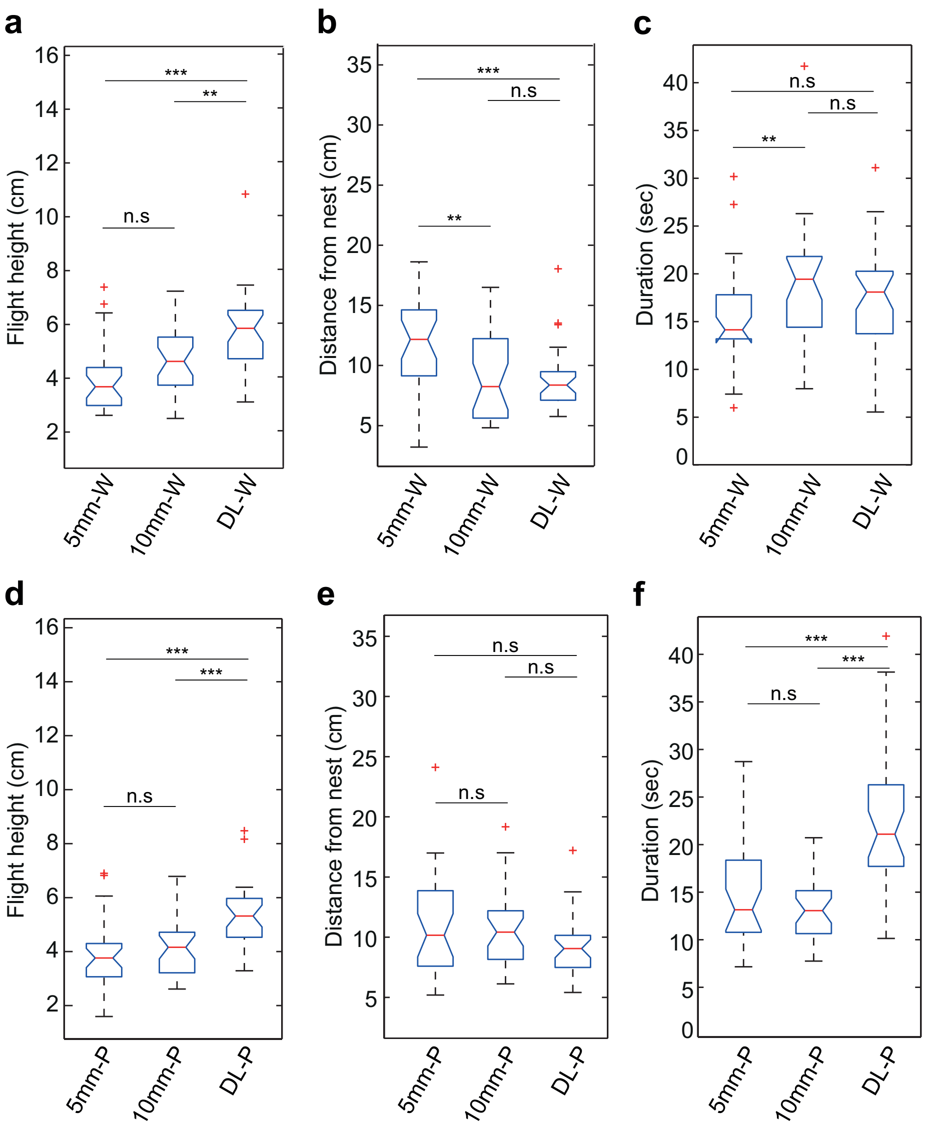

Fig. S1 The effect of ventral optic flow on flight height

Subplot **a-c** shows how bees responded when panoramic optic flow was absent (white walls, W). Subplot **d-f** shows how bees responded when panoramic optic flow was present (dead leaves pattern on the walls, P). The subplots show the bumblebees response when ventral optic flow is only available up until a certain height (limited by the ability of the bees to resolve the pattern on the experimental table). The condition is specified on the x-axis: 5mm = 5 mm check pattern on the experimental table, the ventral spatial texture is not resolvable above 10 cm (5mm-W, n=29; 5mm-P, n=30). 10mm = 10 mm check pattern on the experimental table, the ventral spatial texture is not resolvable above 20 cm (10mm-W, n=29; 10mm-P, n=30). DL = dead leaves pattern on the experimental table, the ventral spatial is resolvable throughout all flight heights (DL-W, n=32; DL-P, n=31). (**a, d**) Average height flown above the surface of the arena. (**b, e**) Average lateral distance from the nest. (**c, f**) Average flight duration. Boxes indicate the extent of the 25%-75% interquartile range, the horizontal line in the box indicates the median, whiskers indicate the full extent of the data and red crosses represent outliers. Black stars indicate the level of significance (Wilcoxon rank-sum): $^{**}P<0.01$, $^{***}P<0.001$. n.s. = not significant ($P>0.025$)

Flight parameter	Ventral optic flow	Panoramic optic flow	Interaction
Flight height	Chi=52.237<br>df=2<br>***p<0.001***	Chi=2.609<br>df=2<br>*p=0.106*	Chi=0.476<br>df=2<br>*p=0.787*
Distance from nest	Chi=19.208<br>df=2<br>***p<0.001***	Chi=0.787<br>df=1<br>*p=0.375*	Chi=3.7<br>df=2<br>*p=0.157*
Duration	Chi=25.220<br>df=2<br>***p<0.001***	Chi=1.467<br>df=1<br>*p=0.226*	Chi=34.183<br>df=2<br>***p<0.001***

Table S1 The effect of ventral and panoramic spatial texture on flight control.
Test results for GLM (gamma, log link function) with ventral (3 levels: 5mm checks, 10 mm checks or dead leaves pattern) and panoramic spatial texture (2 levels: present or absent) as between-subject factors and the measured flight parameters (flight height, flight duration and distance from the nest) as dependent variables. There is a strong effect of ventral spatial texture, as we also find in the analysis of the data presented in the main text of the paper. The significant interaction for flight duration suggests that the duration of flights could potentially be influenced by both ventral and panoramic spatial texture. A speculative interpretation could be that panoramic spatial texture might help the bees to fine-scale flight manoeuvres to increase the time spent flying around the nest, which could enable better learning of the nest position

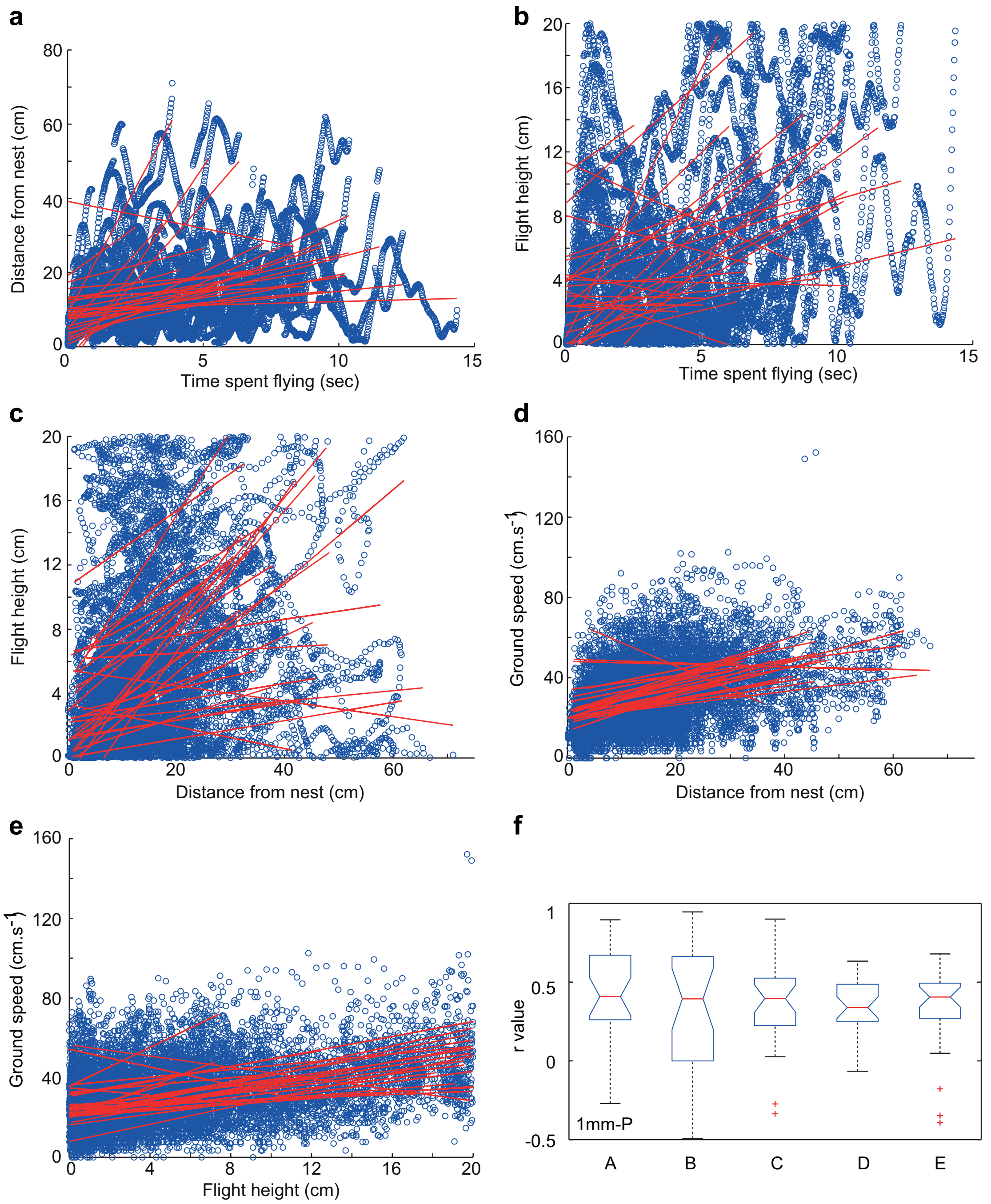

Fig. S2 Flight control in absence of ventral optic flow cues. Subplot **a-f** represents condition 1mm-P (n=29) (1mm check pattern on the experimental platform, and dead leaves pattern on the walls). (**a**) Lateral distance from the nest as a function of time spent flying. (**b**) Flight height as a function of time spent flying. (**c**) Flight height as a function of lateral distance from the nest. (**d**) Ground speed as a function of lateral distance from the nest. (**e**) Ground speed as a function of height flown above the surface of the platform. Red lines indicate a linear regression fit to the data for each flight. (**f**) The associated mean of the Pearson's linear correlation coefficients (r) for condition 1mm-P. Note that A-E correspond to subplots a-e, respectively. Blue boxes indicate the extent of the 25%-75% interquartile range, the red horizontal line in the box indicates the median, whiskers indicate the full extent of the data and red crosses represent outliers
